# An outbreak of *Streptococcus equi *subspecies *zooepidemicus *associated with consumption of fresh goat cheese

**DOI:** 10.1186/1471-2334-6-36

**Published:** 2006-02-27

**Authors:** Markku Kuusi, Elina Lahti, Anni Virolainen, Maija Hatakka, Risto Vuento, Leila Rantala, Jaana Vuopio-Varkila, Eija Seuna, Matti Karppelin, Marjaana Hakkinen, Johanna Takkinen, Veera Gindonis, Kyosti Siponen, Kaisa Huotari

**Affiliations:** 1 National Public Health Institute (KTL), Department of Infectious Disease Epidemiology, Helsinki, Finland; 2 National Veterinary and Food Research Institute (EELA), Helsinki, Finland; 3 National Public Health Institute (KTL), Department of Microbiology, Helsinki, Finland; 4 National Food Agency, Helsinki, Finland; 5 Tampere University Central Hospital, Centre for Laboratory Medicine, Tampere, Finland; 6 Tampere University Central Hospital, Department of Medicine, Tampere, Finland

## Abstract

**Background:**

*Streptococcus equi *subspecies *zooepidemicus *is a rare infection in humans associated with contact with horses or consumption of unpasteurized milk products. On October 23, 2003, the National Public Health Institute was alerted that within one week three persons had been admitted to Tampere University Central Hospital (TaYS) because of *S. equi *subsp.* zooepidemicus *septicaemia. All had consumed fresh goat cheese produced in a small-scale dairy located on a farm. We conducted an investigation to determine the source and the extent of the outbreak.

**Methods:**

Cases were identified from the National Infectious Disease Register. Cases were persons with *S. equi *subsp. *zooepidemicus *isolated from a normally sterile site who had illness onset 15.9-31.10.2003. All cases were telephone interviewed by using a standard questionnaire and clinical information was extracted from patient charts. Environmental and food specimens included throat swabs from two persons working in the dairy, milk from goats and raw milk tank, cheeses made of unpasteurized milk, vaginal samples of goats, and borehole well water. The isolates were characterized by ribotyping and pulsed-field gel electrophoresis (PFGE).

**Results:**

Seven persons met the case definition; six had septicaemia and one had purulent arthritis. Five were women; the median age was 70 years (range 54–93). None of the cases were immunocompromized and none died. Six cases were identified in TaYS, and one in another university hospital in southern Finland. All had eaten goat cheese produced on the implicated farm. *S. equi *subsp.* zooepidemicus *was isolated from throat swabs, fresh goat cheese, milk tank, and vaginal samples of one goat. All human and environmental strains were indistinguishable by ribotyping and PFGE.

**Conclusion:**

The outbreak was caused by goat cheese produced from unpasteurized milk. Outbreaks caused by *S. equi *subsp. *zooepidemicus *may not be detected if streptococcal strains are only typed to the group level. *S. equi *subsp. *zooepidemicus *may be a re-emerging disease if unpasteurized milk is increasingly used for food production. Facilities using unpasteurized milk should be carefully monitored to prevent this type of outbreaks.

## Background

*Streptococcus equi *subspecies *zooepidemicus *belongs to the betahemolytic Group C streptococci, which can cause disease both in animals and humans [1]. *S. equi *subsp. *zooepidemicus *may be found in the nasopharynx, on the tonsils, in the respiratory tract, and on the genital mucous membranes of healthy horses and cattle [2]. It is an important cause of respiratory tract infections in foals and young horses, and it is associated in uterine infections in mares [3]. It has also been associated with a wide variety of infections, including mastitis, in pigs, sheep, cows, goats, and several other mammalian species [4-6]. Human infections are rare; the clinical presentations include pharyngitis, septicemia, meningitis, purulent arthritis and endocarditis. Poststreptococcal glomerulonephritis has also been described in connection with human infections [7,8]. Outbreaks with high mortality have been reported [9,10]. The source of human infection is often traced back to contact with domestic animals, especially horses, or ingestion of unpasteurized milk or milk products [11].

All invasive streptococcal infections are notifiable to the National Infectious Disease Register (NIDR) in Finland (population 5.2 million). Between 1998 and 2002, only three cases of invasive *S. equi *subsp. *zooepidemicus *infection were notified to NIDR. In addition, during the same time period, from 7 to 11 cases of invasive Group C streptococcal infections per year were notified. In these cases the streptococcal strains were only typed to the group level.

On October 23, 2003, the National Public Health Institute (KTL) was notified that three patients with *S. equi *subsp. *zooepidemicus *septicemia had been admitted to Tampere University Hospital from October 14 to October 19, 2003. Epidemiological, microbiological and environmental investigations were conducted to determine the source and the extent of the outbreak.

## Methods

### Epidemiological investigation

Cases were identified from the NIDR. A case was defined as a person with an illness in which *S. equi *subsp. *zooepidemicus *was isolated from a normally sterile site from September 15 to October 31, 2003. In addition to cases notified to NIDR, the clinical microbiology laboratories in the country's 21 health care districts were requested to type to species level all group C streptococcal strains isolated from a normally sterile site between September 15 and October 31.

To determine the source of the infection, all cases were interviewed by telephone by using a standard questionnaire. Cases were asked about underlying illnesses, onset and symptoms of current illness, contact with farm and other animals, and consumption of goat cheese and unpasteurized milk products. In addition, clinical information was extracted from patient charts, including results of laboratory tests and antimicrobial treatment. In outbreak investigations in Finland, ethics committee approval is not required. During the telephone interview, a verbal informed consent was obtained from the patients.

### Environmental investigation

Initial interviews suggested that goat cheese manufactured in a small-scale dairy on a farm was associated with the cases. Samples from cheeses (n = 11) produced on the farm on October 20, October 23, and October 27 were collected from retail stores and the farm. The farm was inspected on October 28 by local authorities, and milk samples were collected from the farm's milk tank (n = 1) and from all goats (n = 34) in lactation. In addition, nasal swab samples were taken from 10 goats, and vaginal samples (n = 34) from all goats in lactation. Water samples (n = 2) from the farm's borehole well were taken, as well as environmental swabs (n = 22) from the dairy and the barn. Milk samples were also collected from goats (n = 48) and milk tank (n = 1) on another farm delivering milk to the dairy. Throat swabs were taken from the two persons who manufactured cheese in the dairy.

### Laboratory methods

Clinical microbiology laboratories isolated *S. equi *subsp.*zooepidemicus *by using routine microbiological methods [12]. Samples from the cheese, the environment, and the goats were examined at the National Veterinary and Food Research Institute (EELA) [13]. Cheese samples were diluted in peptone water until 10^-7^, streaked onto bovine blood agar plates and incubated anaerobically and aerobically at 37°C. Vaginal and nasal swabs from goats were streaked onto bovine blood agar plates and onto Edwards agar and incubated anaerobically and at CO_2 _(5%) atmosphere at 37°C. Milk samples were streaked onto bovine blood-aesculin agar plates. Environmental swab samples were enriched in Todd-Hewitt broth containing nalidixic acid (15 mg/l) and colistin (10 mg/l) [8] and streaked onto Edwards agar and bovine blood-aesculin agar plates supplemented with and without nalidixic acid (15 mg/l) and colistin (10 mg/l). Beta hemolytic pin point colonies were identified using conventional methods: gram staining, catalase test, the Lancefield group C using a commercial latex test Streptex (Remel Europe, Dartford, UK), and biochemical characterization using API 20 Strep and Rapid ID32 (bioMérieux, Marcy-l'Étoile, France). Susceptibility to antimicrobial agents was performed by using a commercial microdilution method VetMIC™ (SVA, Uppsala, Sweden).

At KTL, the species identification was confirmed by biochemical tests and the strains were ribotyped as described previously by using *Hind*III and *Eco*RI as the restriction enzymes [14,15]. The strains were genotyped at EELA by using pulsed-field gel electrophoresis (PFGE). DNA isolation was performed as described previously [16], and 40 U of *Sma*I and 20 U of *Apa*I (New England Biolabs, Beverly, USA) were used for digestion. Electrophoresis conditions were as follows: 120°, 14°C, 6 V/cm and switch times 5–40 s, 20 h and 1–15 s, 18 h, respectively. For testing discriminatory power of the typing methods five *S. equi *subsp.*zooepidemicus *strains: one human clinical isolate from sporadic case KTL 03V05424, isolates from bovine mastitis EELA179, horse EELA BA6997/1, ATCC 700400 and EELA tyyp656 (DEFRA intercalibration) were used as control strains. A one-band difference in PFGE profiles and ribopatterns was considered to represent a new type.

## Results

### Epidemiology and clinical features

Altogether seven cases were identified, six of which were from the Tampere University Hospital and one from the Helsinki University Hospital. In one case, *S. equi *subsp.*zooepidemicus *was identified after the microbiology laboratories had typed the invasive group C streptococcal isolates further to species level. Two cases were men and five were women. The median age was 70 years (range 54–93 years). The median incubation period from eating goat cheese to onset of symptoms was two days (range 0–12). Six cases had septicemia, and one had purulent arthritis (see additional file 1). Whether this case also had septicemia is unknown, because blood cultures were not taken. None of the cases had malignant diseases, or severe immunosuppression. All cases were treated with antimicrobial therapy. After the blood culture results were obtained, four patients received intravenous penicillin either alone or combined with aminoglycosides, two patients continued with initial cefuroxime treatment, and one who had allergy to penicillin received levofloxacin combined with rifampicin. All cases recovered without complications, and were discharged after a median hospitalization of 14 days (range 6–32 days).

All cases had eaten goat cheese during October 2003. Six cases reported that the goat cheese they had eaten was a brand that was manufactured in a small-scale dairy located on a farm in the vicinity of Tampere. The remaining case had eaten goat cheese, but did not remember the brand. However, the cheese was purchased from a retail store that was selling goat cheese manufactured on the implicated farm.

### Microbiological investigations

Blood cultures of six cases and joint fluid culture of one case yielded morphologically identical group C streptococci, which also shared identical biochemical profiles and were thus identified as *S. equi *subsp.*zooepidemicus *in the clinical laboratories. Similarly identified *S. equi *subsp. *zooepidemicus *was isolated from a bulk milk sample, samples from cheese (dilutions 10^-1 ^and 10^-2^) manufactured on October 23, and two consecutive vaginal samples from one goat out of 34 animals examined. None of the ten nasal swabs from the goats yielded *S. equi *subsp.*zooepidemicus*, neither did the milk samples from individual goats on the farm nor the environmental and water samples. Milk samples from goats and bulk milk on the other farm producing milk to the dairy were negative. Throat samples from the two persons who manufactured goat cheese in the dairy also yielded *S. equi *subsp. *zooepidemicus*. These persons had suffered from symptoms of pharyngitis. All strains had decreased susceptibility to clindamycin (MIC = 0.5).

**Figure 1:**
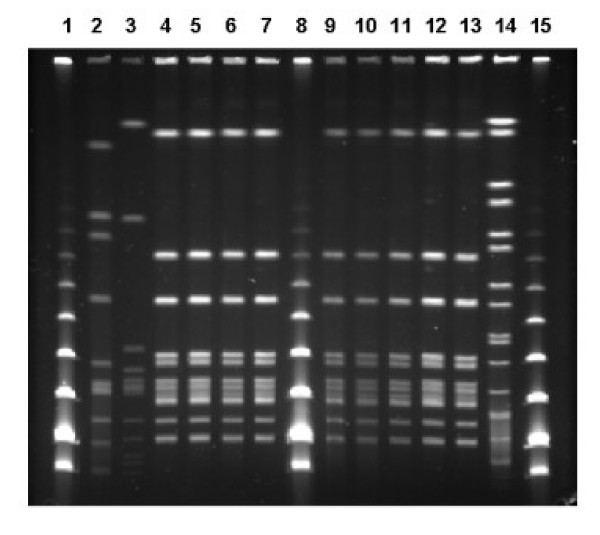
PFGE patterns (*SmaI*) of *S. equi *subsp. *zooepidemicus *isolates. Lane 2, control strain *S. equi *ssp. *zooepidemicus *EELA 179; lane 3, human clinical isolate from a sporadic case; lanes 4–6, human clinical isolates from the outbreak; lane 7, throat isolate from foodhandler; lanes 9–10, isolates from goat cheese; lane 11, bulk milk from tank; lanes 12–13 vaginal secretion of the goat; lane 14, *Salmonella *Braenderup CDC H9812; lanes 1, 8 and 15, Low Range marker (NEB).

**Figure 2:**
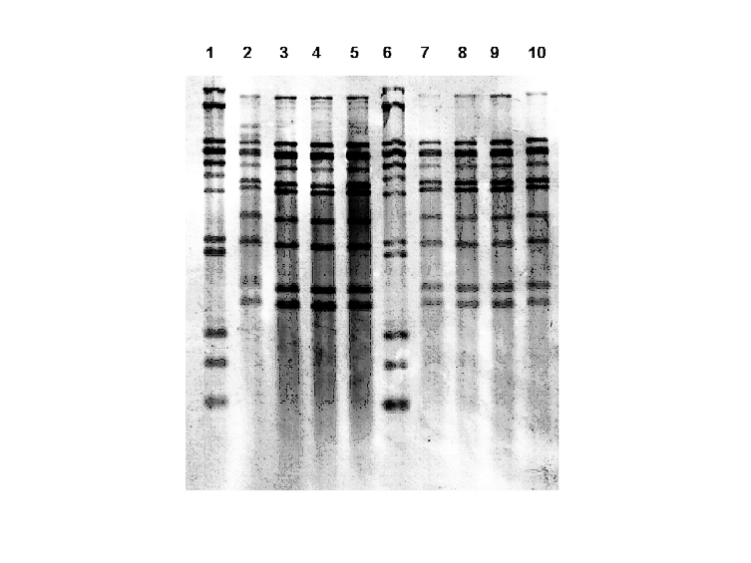
Ribotyping patterns with the restriction enzyme *Hind*III of *S. equi *subsp. *zooepidemicus *isolates from four different human patients (lanes 2–5), goat cheese (lanes 7–8), bulk milk (lane 9) and vaginal secretion of the goat (lane 10). Molecular weight marker (lanes 1 and 6).

Ribotyping and PFGE (*Sma*I and *Apa*I) patterns of the isolates associated with the outbreak (human clinical isolates, throat isolates from dairy workers, isolates from cheese, raw milk and goat) were indistinguishable (Figures 1 and 2). The PFGE and ribotyping patterns of the control strains were clearly different from each other, and also from the outbreak strain pattern.

### Environmental investigation and trace-back

The farm had 40 goats producing milk that was only used for cheese production. In addition, the dairy on the farm received about 200 liters milk per week from another goat farm for cheese production. Milk from the farms was not mixed but used separately for the production. However, labeling in the cheese packages did not indicate the origin of the milk. Altogether, the dairy used about 30,000 liters of goat milk per year. The dairy manufactured two types of cheeses, fresh and feta cheese. During manufacture of fresh cheese, the milk was warmed to 32°C, while during manufacture of feta cheese, the milk was heated to 75°C. Microbiological quality of the milk, including counts of heterotrophic bacteria and staphylococci, was monitored once per month. Cheese samples were routinely tested 11 times a year for listeria, salmonella, total heterotrophic bacteria, staphylococci and coliform bacteria. Staphylococci had been detected in some of these samples, but the goats had not presented any signs of mastitis. Goats on the farm had previously had abortions, but although samples were sent for examination the cause of these had remained unknown.

### Control measures

Cheese manufactured on the farm was distributed to 14 retail stores located in the county of Pirkanmaa. The producer transported cheeses herself to the retail stores and collected at the same time cheeses that were not sold before the expiry date. Cheeses were also distributed to one spa in the same county and one retail store in a neighboring county. All cheeses produced on the farm were recalled from retail stores on October 28, 2003. At the time of recall, cheeses produced on October 20, October 23, and October 27 were still available in the retail stores. Altogether 53.5 kg cheese was collected from the retail stores, mainly from the batch produced on October 27. Of the 15 kg batch produced on 23 October, 2.2 kg were collected back. Samples from this batch yielded *S. equi *subsp. *zooepidemicus*. Production of cheese in the dairy was stopped on October 28, and use of pasteurized milk was required for restart of production. The goat found to be carrier of *S. equi *subsp. *zooepidemicus *was slaughtered to prevent further contamination of milk.

## Discussion

This *S. equi *subsp.*zooepidemicus *outbreak was caused by fresh goat cheese manufactured from unpasteurized milk in a small dairy on a farm. Six of seven cases had eaten fresh goat cheese manufactured in this dairy, and also the seventh case had probably eaten the same cheese. Human, cheese, bulk milk and goat samples yielded *S. equi *subsp.*zooepidemicus*. Molecular typing with two different methods showed indistinguishable patterns for all outbreak associated strains, providing strong evidence that goat cheese was the source of infections.

All cases with invasive *S. equi *subsp.*zooepidemicus *disease recovered in this outbreak. In several other outbreaks, the case fatality proportion (CFP) has been substantial. Among 11 patients hospitalized in Hong Kong because of septicemia from 1982 to 1986, two died (CFP 22%) [10]. Two persons of sixteen (CFP 12%) died in an outbreak associated with homemade cheese in New Mexico in 1983 [17]. In another outbreak affecting 11 persons caused by unpasteurized milk in England in 1984, seven patients died (CFP 64%) [9]. In a series of three cases, two of which were septicemias, in England in 1985, one patient died [18]. Single cases of invasive infection with variable outcomes have been reported from several countries [19-26]. Although the median age of cases in this outbreak was high, none of them had severe underlying illnesses, which may explain that all of them recovered. In addition, after previously reported outbreaks from the 1980s, substantial development has taken place in the treatment of severely ill patients. The clinical features of illness in this outbreak were severe, however, which is demonstrated by the long hospitalization required for most cases.

The cases with invasive disease likely represent only a small proportion of all persons infected in this outbreak, because after the outbreak became public in the media, several persons reported that they had suffered from pharyngitis after having eaten the implicated goat cheese. Of the contaminated batch from October 23, 13 kg were sold, which means that probably tens of people were exposed to cheese from this batch. In addition, our cases had eaten cheese from batches manufactured earlier in October, indicating that also other batches have been contaminated.

Invasive group C streptococcal infections are not common in Finland. In most clinical laboratories, the strains are routinely typed only to the group level. Identification of the strains to species level would better enable public health professionals to detect outbreaks. In addition, even in individual cases, typing to species level might help in determining the probable source of infection. Molecular typing in this outbreak provided strong evidence that cheese from the implicated dairy was the source of the outbreak. Because the outbreak had substantial economic consequences to the manufacturer, it was important to get as strong evidence as possible about the source of these infections, also by using two types of molecular fingerprinting methods.

The two persons manufacturing goat cheese may have acquired their infection from contaminated milk or cheese. However, the owner might also have acquired the infection through direct contact with goats as she milked the animals and took care of them. Transmission of *S. equi *subsp. *zooepidemicus *through direct animal contact has been described in several reports [7,19,22]. The other food handler participated only in the manufacture of cheese and might have become infected through the manipulation of contaminated cheese. Also person-to-person transmission might have occurred, because symptomless food handlers can carry the organism in the throat [8].

*S. equi *subsp.*zooepidemicus *is a normal commensal of mucous membranes of a variety of animal species, especially horses. It is not known to be a common cause of genital infections in goats. Some cases of mastitis and meningitis have been reported [27,28]. The prevalence of carrier state is not known. In the present case, the *S. equi *subsp.*zooepidemicus *-positive animal was in heat during the first sampling, and abundant growth of *S. equi *subsp.*zooepidemicus *was detected on the primary plates. The vaginal discharge might have contaminated the udder and the milk during milking. In the second sample the growth was sparse, and could easily have been overlooked. This indicates that the number of organisms excreted may vary, and may be difficult to detect.

The isolation of the bacterium from cheese samples was performed without using a selective medium which might have weakened the sensitivity of the analysis as the background flora was abundant. The dilution of the samples, however, enhanced the detection of the organism. The vaginal and nasal samples therefore were not heavily contaminated and the only goat with an isolation of *S. equi *subsp. *zooepidemicus *had an abundant growth of the organism.

Pasteurization is effective in preventing transmission of bacterial infections through milk products, and nearly all milk products in Finland are manufactured of pasteurized milk. Several outbreaks reported in the literature have been associated with unpasteurized milk, including salmonellosis, campylobacteriosis, listeriosis, and *S. equi *subsp. *zooepidemicus *infections [11]. After pasteurization of milk became common, some of these infections, including *S. equi *subsp. *zooepidemicus *have become rare. If unpasteurized milk is used for manufacture of foods, the facility should be monitored carefully, including frequent microbiological sampling of the lactating animals and the products. The spectrum of the microbes examined routinely in Finland may not be sufficient for these facilities, but it should be extended to include *S. equi *subsp. *zooepidemicus *or beta-hemolytic streptococci as an indicator group. Because delicacy products manufactured of unpasteurized milk, like the goat cheese in this outbreak, may become more common, the importance of rigorous routine control measures should be emphasized.

## Conclusion

The investigation provided strong evidence that this *S. equi *subsp. *zooepidemicus *outbreak was caused by fresh cheese produced from unpasteurized milk. Seven patients had severe invasive illness, but none died. Outbreaks caused by *S. equi *subsp. *zooepidemicus *may not be detected, if streptococcal strains in invasive infections are only typed to the group level. Facilities using unpasteurized milk for food production should be carefully monitored to prevent this type of outbreaks.

## Competing interests

The author(s) declare that they have no competing interests.

## Authors' contributions

MKu supervised the epidemiological investigation and drafted the manuscript.

EL participated in the investigation of food samples and contributed to the writing of the article.

AV carried out the ribotyping and contributed to the writing of the article.

MHat coordinated sampling of foods and contributed to the writing of the article.

RV detected the outbreak, made the microbiological diagnosis, and contributed to the writing of the article.

LR carried out the PFGE and contributed to the writing of the article.

JVV interpreted the ribotyping findings and contributed to the writing of the article.

ES participated in the analysis of goat samples and contributed to the writing of the article.

MKa made the initial interviews of patients and commented different versions of the article.

MHak participated in the analysis of food samples and contributed to the writing of the article.

JT participated in the epidemiological investigation and commented different versions of the article.

VG participated in the analysis of goat milk samples and commented different versions of the article.

KS commented different versions of the article.

KH interviewed the patients, collected clinical information, and contributed to the writing of the article.

## Pre-publication history

The pre-publication history for this paper can be accessed here:



## Supplementary Material

Additional File 1 Clinical and laboratory characteristics of seven cases with invasive *S. equi *subsp. *zooepidemicus *infection, Finland, October 2003. Click here for file
