# Supplementary material for: An outbreak of Streptococcus equi subspecies zooepidemicus associated with consumption of fresh goat cheese
Source: BMC Infect Dis. 2006 Feb 27;6:36. doi: 10.1186/1471-2334-6-36 (PMC1413536; doi:10.1186/1471-2334-6-36)
Supplement: Additional File 1 — Clinical and laboratory characteristics of seven cases with invasive S. equi subsp. zooepidemicus infection, Finland, October 2003. [file 1471-2334-6-36-S1.doc]

Table 1. Clinical and laboratory characteristics of seven cases with invasive *S. equi* subsp. *zooepidemicus* infection, Finland, October 2003.

| **Age** | **Sex** | **Eaten goat cheese** | **Onset of illness** | **Underlying illness** | **Clinical symptoms** | **Temperature at admission**  **(ºC)** | **WBC**  **Adm^#^**  **Highest**  **(x10^9^/l)** | **CRP**  **Adm^#^**  **Highest**  **(mg/l)** | **S. equi subsp. zooepidemicus isolated from** | **Antimicrobial treatment*** | **Duration of hospitalization**  **(days)** |
| --- | --- | --- | --- | --- | --- | --- | --- | --- | --- | --- | --- |
| 57 | Female | 10.10.2003 | 12.10.2003 | Hypertension  Coeliacia | Fever  Arthralgia | 37.6 | 15.3  16.2 | 346  346 | Blood | 1. Cefuroxime i.v  2. Penicillin i.v.  3. Penicillin p.o. | 21.10-5.11.2003  (15 days) |
| 71 | Male | 11.10.2003 | 13.10.2003 | None | Fever  Arthralgia | 38.2 | 14.4  17.5 | 258  258 | Blood | 1. Cefuroxime i.v  2. Penicillin i.v. + gentamycin  3. Penicillin p.o. | 19-31.10.2003  (12 days) |
| 64 | Female | 11.10.2003 | 13.10.2003 | None | Fever  Cough  Arthralgia  Vomiting | 40.3 | 15.2  15.2 | 180  202 | Blood | 1. Cefuroxime  2. Cephalexin p.o. | 14-20.10.2003  (6 days) |
| 54 | Female | 11.10.2003 | 13.10.2003 | Osteoporosis | Fever  Vomiting  Lymphadenopathia | NR** | 9.4  9.4 | <5  172 | Blood | 1. Roxithromycin p.o  2. Cefuroxime i.v  3. Penicillin p.o. | 14-22.10.2003  (8 days) |
| 69 | Male | 4-5.10.2003 | 16.10.2003 | Hypercholesterole-mia | Fever | 37.3 | 8.0  14.1 | 35  73 | Blood | 1. Cefuroxime i.v 2. Penicillin i.v  3. Penicillin p.o | 31.10-13.11.2003  (14 days) |
| 82 | Female | 18.10.2003 | 19.10.2003 | Diabetes mellitus type 2  Coronary heart disease  Knee arthrosis | Fever  Swelling, redness and pain of left knee | 38.4 | 14.7  14.7 | 305  305 | Joint fluid (knee) | 1. Clindamycin i.v 2. Levofloxacin i.v + rifampicin | 26.10-27.11.2003  (32 days) |
| 93 | Female | 27.10.2003 | 27.10.2003 | Hypertension | Fever  Myalgia  Vomiting  Headache | 40.4 | 19.0  19.0 | 77  295 | Blood | 1. Cefuroxime i.v  2. Penicillin i.v + tobramycin  3. Penicillin p.o | 31.10-14.11.2003  (15 days) |

* in order of administration

** NR=not reported

# at admission
